# Supplementary material for: Impact of Parental Knowledge and Beliefs on HPV Vaccine Hesitancy in Kenya—Findings and Implications
Source: Vaccines (Basel). 2022 Jul 26;10(8):1185. doi: 10.3390/vaccines10081185 (PMC9332201; doi:10.3390/vaccines10081185)
Supplement: Supplementary file 1 [file vaccines-10-01185-s001.zip › vaccines-1796426-supplementary.pdf]

SUPPLEMENTARY MATERIAL ON IMPACT OF PARENTAL KNOWLEDGE AND BELIEFS ON HPV VACCINE HESITANCY IN KENYA; FINDINGS AND IMPLICATIONS

INTRODUCTION

This section contains additional data that supplements the study on parental knowledge and beliefs towards HPV vaccination.

S1. Sources of Parental Information on the HPV Vaccine and determinants

Parental sources of information of HPV vaccine are summarized in Figure S1.

Figure S1: Sources of Parental Information on the HPV Vaccine

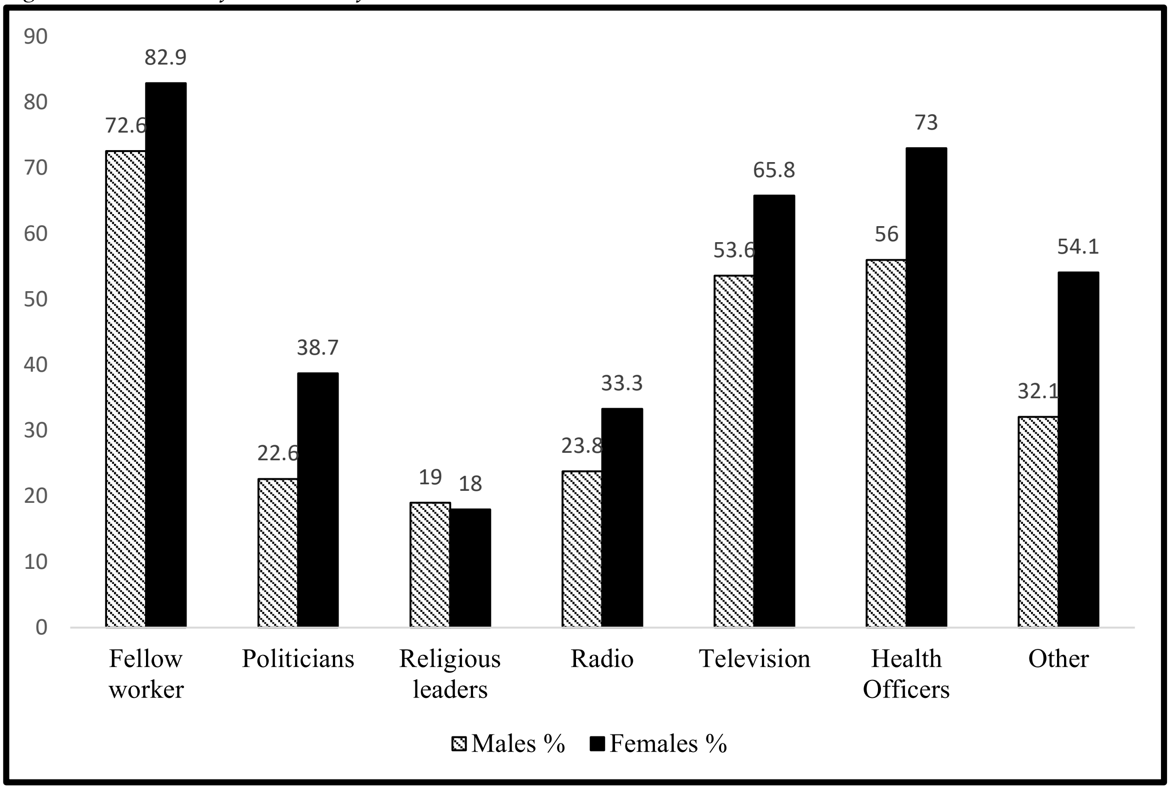

NB: Others* social media

S2. Knowledge about the Human Papilloma Virus (HPV)

Figure S2. summarizes proportions of participants who had knowledge about various aspects of HPV infection.

Figure S2: Gender differences in knowledge about the Human Papilloma Virus (HPV)

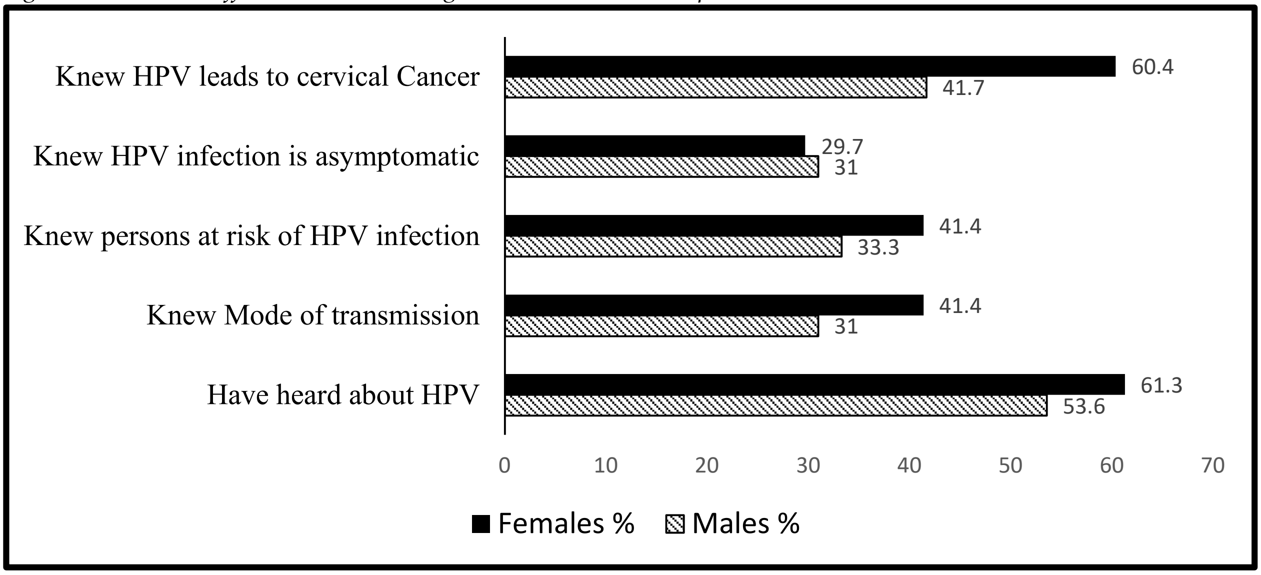

S3. Knowledge about Cervical Cancer

The parental knowledge about cervical cancer including potential prevention measures is summarized in Figure S3.

Figure S3: Gender differences in knowledge about Cervical Cancer.

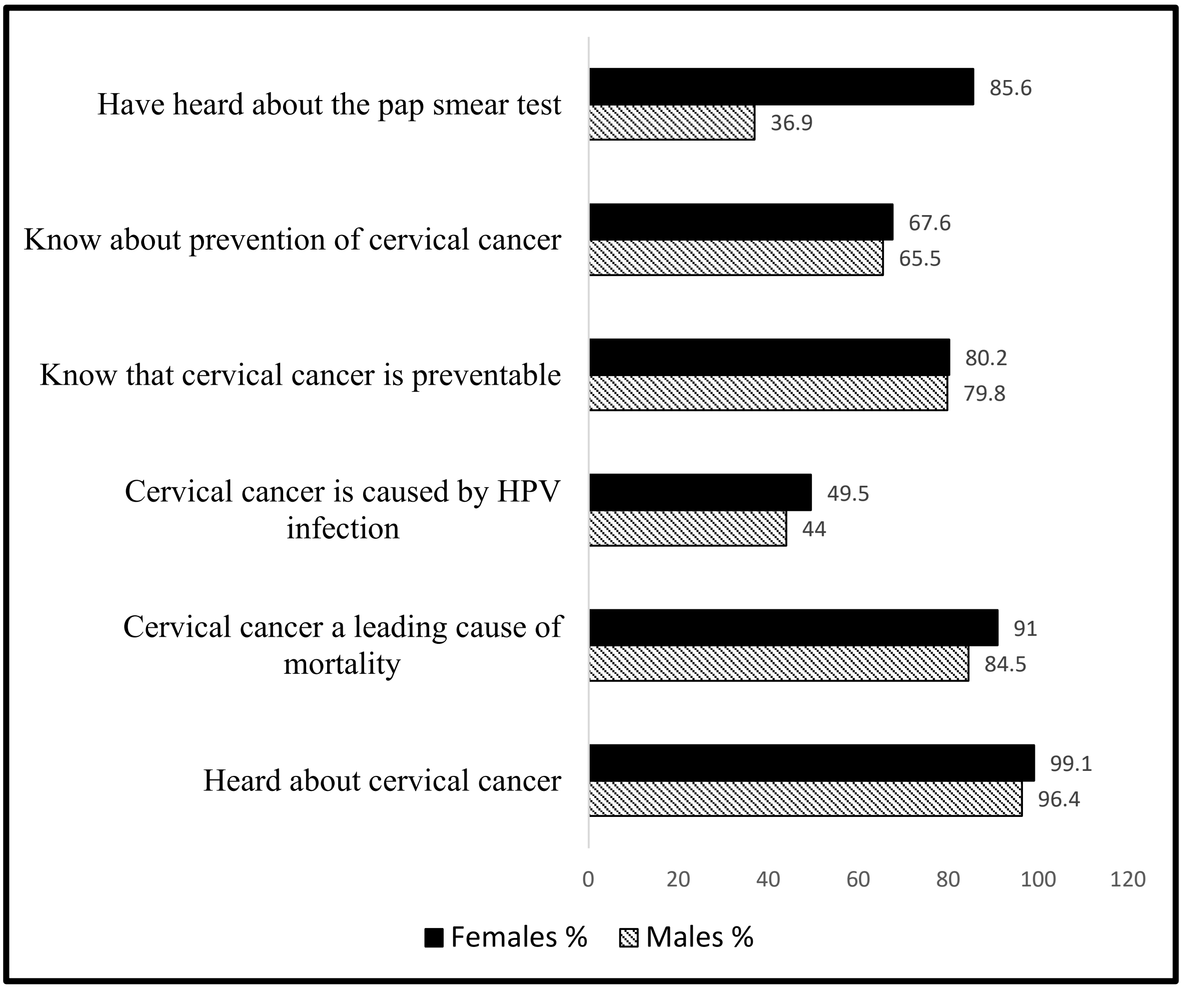

S4. The Interaction Effects of Age and Gender on Knowledge Score

A significant finding was a statistical interaction between age and gender, as noted in Table 2 (p=0.033). On bivariable analysis, age did not seem to affect knowledge. This can be attributed to the fact that its effect was modified by gender. However, on controlling for the modifying effect of gender, age had a significant effect on participant's knowledge levels. The modifying effects of gender across the ages of the participants are illustrated in Figure S4.

Figure S4: The effects of Age and Gender on Knowledge Score

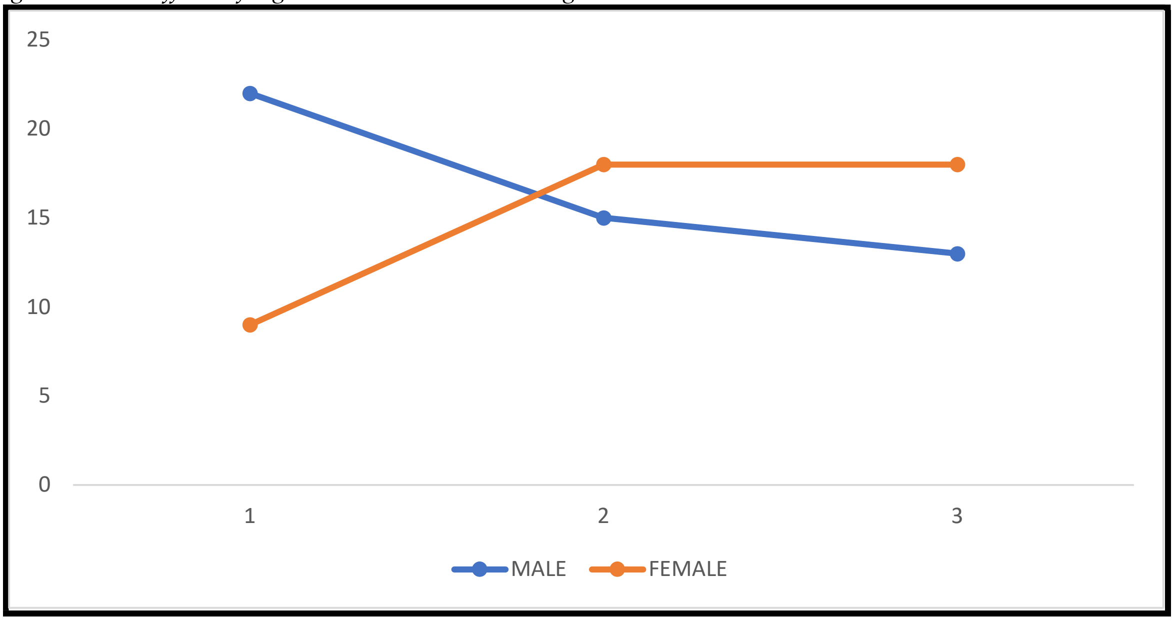

NB: 1 represents age 18-30 years; 2 represents age 31-40 years; 3 represents = 40 years and above

Amongst young parents aged between 18-30 years, males had a higher knowledge score than the females. After 31 years, the females had a higher knowledge score than the males. Older males aged above 40 tended to score worse than their female counterparts. Overall, there was a negative association between age and knowledge score in males and a positive association among females.

S5. Parental views on knowledge empowerment

The findings regarding possible knowledge empowerment by parents are summarized in Figure S5.

Figure S5: Parental desire for more knowledge

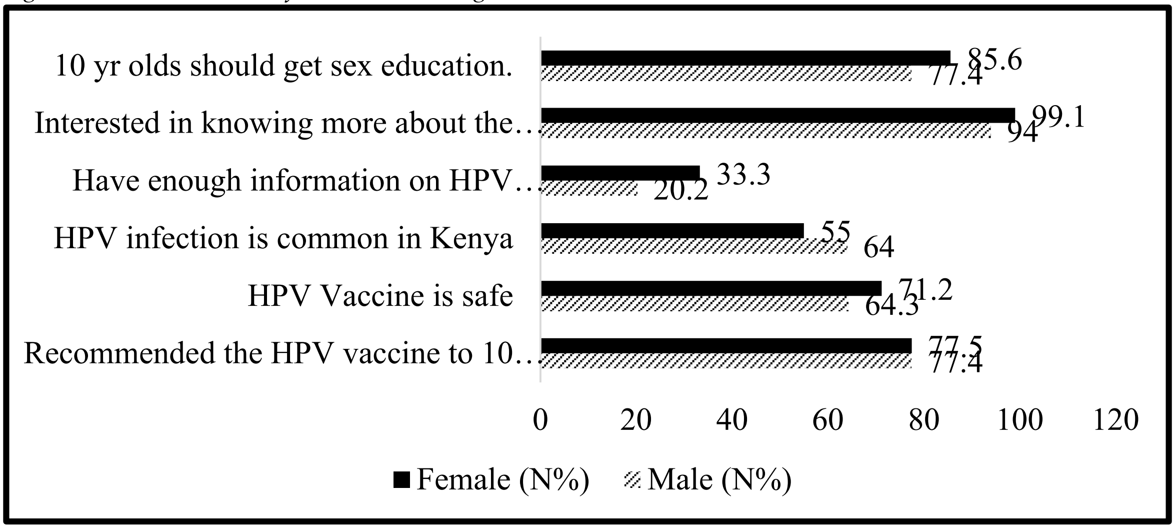

S6. Parental Beliefs on the Vaccine

The respondents' beliefs on the HPV vaccine were summarized on Table S1.

Table S1: Beliefs about the HPV Vaccines

**Belief held by the parent**	**Males** **N (%)**	**Females** **N (%)**	**Total (overall cohort)** **N (%)**	**P value**
Child is at risk of HPV infection	58 (69.0%)	79 (71.1%)	137 (69.70%)	0.472
HPV infection is severe	60 (71.4%)	74 (63.9%)	134 (68.70%)	0.790
HPV vaccine is effective	48 (57.1%)	73 (65.7%)	121 (62.10%)	0.321

S7. Parental Willingness to have their Children Vaccinate

Figure S6: HPV vaccination willingness

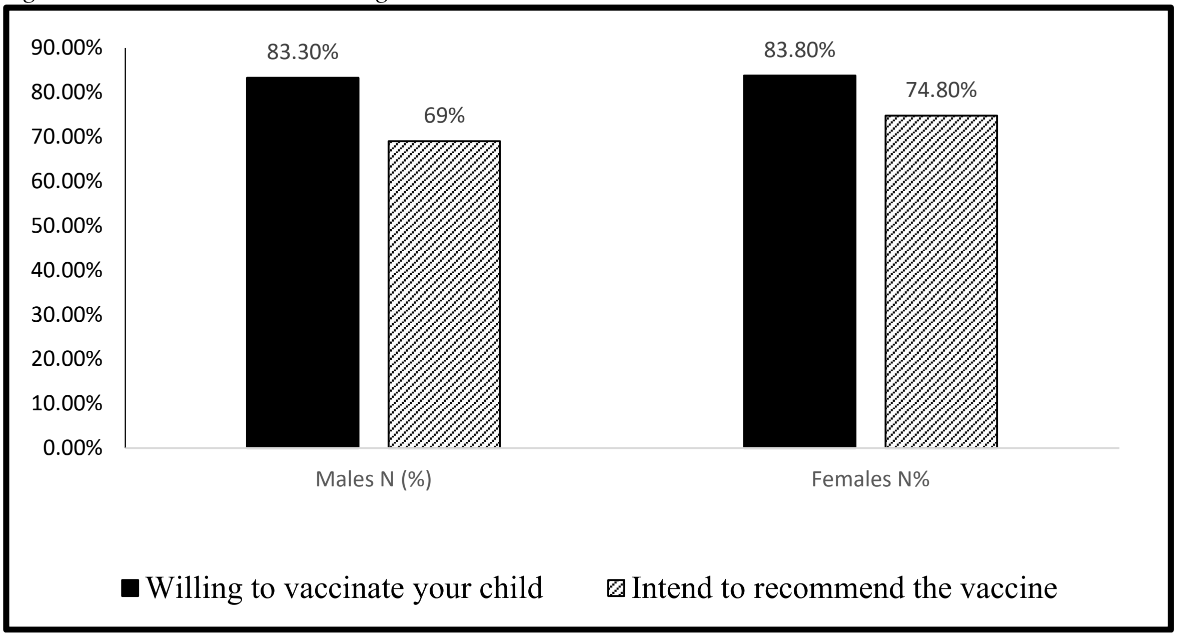

S8. Reasons for HPV vaccination acceptance

Figure S7: Reasons for HPV vaccination acceptance

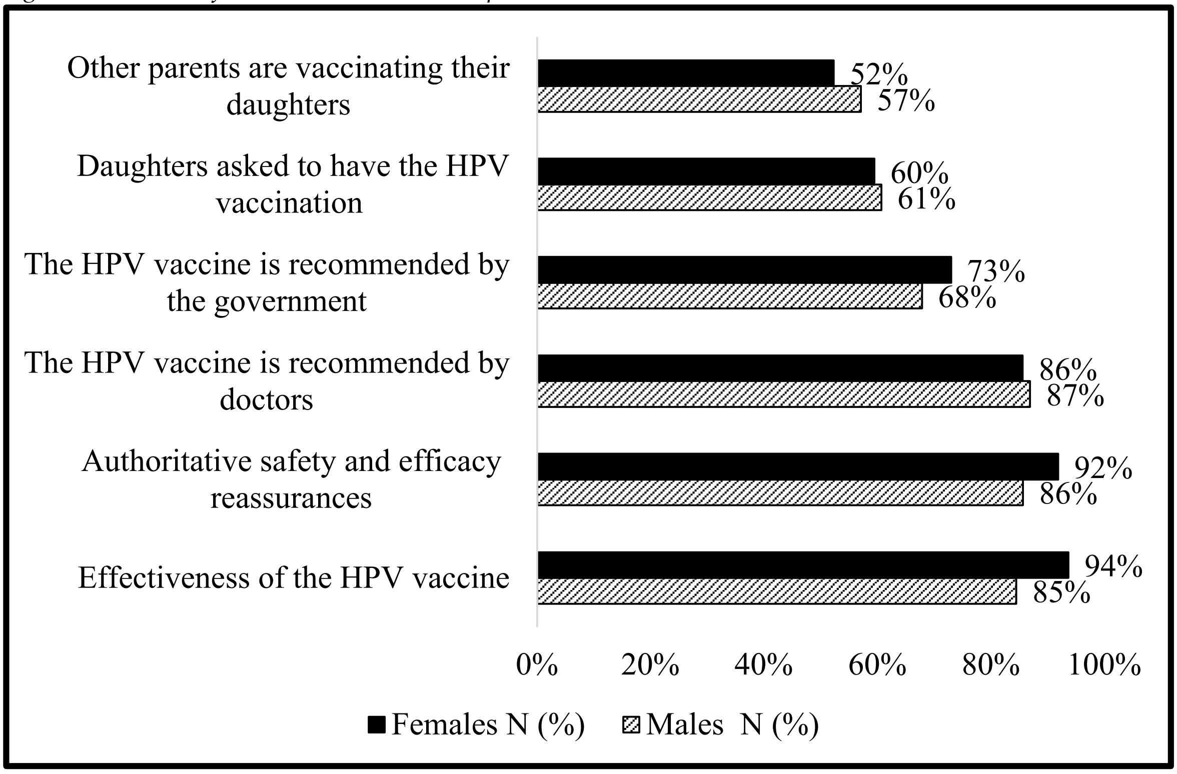

Text S1. Questionnaire

STUDY TITLE: DETERMINANTS OF HPV VACCINE HESITANCY AMONG PARENTS ATTENDING KENYATTA NATIONAL HOSPITAL OUTPATIENT CLINICS

Guidelines

1. Give your answers by appropriately responding in the blank spaces provided. This will involve either writing the responses or circling the appropriate choice as the questions will require.
2. Feel free to ask for clarification whenever in need.

SECTION 1: Sociodemographic characteristics.

1. What is your age?____________Years. Date of Birth (Year): _______________

**Age category (Years)**	**(Tick)**	**Code**
18-30		1
31-40		2
Above 40		3

2. Sex:

**Category**	**(Tick)**	**Code**
Male		1
Female		0

3. What is your Occupation:

**Category**	**(Tick)**	**Code**
Formal employment		1
Self-employed		2
Other (specify)……………….		3

4. What is your Level of education so far:

**Category**	**(Tick)**	**Code**
I have no formal education		1
Primary level		2
Secondary level		3
Tertiary		4

5. What is your marital status?

**Category**	**(Tick)**	**Code**
Married		0
Single (Divorced/never married/ widow/widower)		1

6. What is your religion?

**Category**	**(Tick)**	**Code**
Christian		1
Muslim		2
Others ((specify):		3

7. If yes?

**Category**	**(Tick)**	**Code**
Yes, boys only		1
Yes, girls only		2
Yes, boys and girls		3

8. What is /are the age/ages of the girls…………………

What is the estimated average age of children (girls) in your family?

	Category	(Tick)	Code
**9.**	8 years of younger		0
**10.**	9-11 years		1
**11.**	12-14 years		2
**12.**	15 years or older		3
**13.**	Not Applicable		4

SECTION 2: Awareness and knowledge of the HPV vaccine.
HPV

14. Have heard about HPV infection?

Category	(Tick)	codes
Yes		1
No		0

What is the mode of transmission of HPV? (Tick any/all that apply)

	Category	(Tick)	Yes(codes)	No (codes)
**15.**	Physical content		1	0
**16.**	Aerosol/Air droplet		1	0
**17.**	Sexual intercourse		1	0
**18.**	Other (specify):		1	0

Knowledge about mode of transmission (correct =1, incorrect = 0)

Which of the following persons can be infected by HPV

		Tick	Yes=1	No = 0	I don't know =2
**19.**	Male		1	0	
**20.**	Female		1	0	
**21.**	Both		1	0	
**22.**	I don't know				

Knowledge about persons who can be infected by HPV (correct =1, incorrect = 0)

23. Is everyone infected with HPV going to have symptoms

(**Yes =1, No= 0, I don't know=2)**

- Yes
- No
- I don't know

24. Will Infection with HPV lead to cervical cancer:

(**Yes =1, No= 0, I don't know=2)**

- Yes
- No
- I don't know.

25. How can genital HPV infection be prevented?

……………………………………………………………………………………….

<u>CERVICAL CANCER</u>

26. Have heard about cervical cancer?

**Category**	**(Tick)**	**codes**
Yes		1
No		0

27. Is cervical cancer one of the leading causes of cancer deaths in women in Kenya? (**Yes =1, No= 0, I don't know=2)**

- yes
- no
- I don't know

28. Is cervical cancer caused by the HPV infection? (**Yes =1, No= 0, I don't know=2)**

- Yes
- No
- I don't know

29. Is cervical cancer preventable? (**Yes =1, No= 0, I don't know=2)**

- Yes
- No
- I don't know

If yes, how can cervical cancer be prevented?

	**Categories**	**Tick here**	**Yes (code)**	**No (codes)**
**30.**	Pap smear		1	0
**31.**	Vaccination		1	0
**32.**	Abstinence		1	0
**33.**	Condom use		1	0
**34.**	I don't know		1	0

35. Have you heard about the pap smear test? (**Yes =1, No= 0, I don't know=2)**

- Yes
- No

HPV VACCINE

What is the HPV vaccine used for? (Yes =1, No = 0, I don't know=2)

36. Prevention of HPV infection

- Yes
- No

37. Prevention of cervical cancer

- Yes
- No

38. Prevention of genital warts

- Yes
- No

39. What is /are the age group of your girl children

…………………………………………………………

40. Which ages is/are eligible for the HPV vaccine

	**Tick**	**code**

26 and below		1
Above 26		0
I don't know		2

41. Are you aware that all girls aged 10 years are being offered a Human Papilloma Virus (HPV) vaccine? **(Yes =1, No = 0, I don't know=2)**

- Yes
- No
- I don't know

If YES, how did you hear about it? (**Tick all that apply)**

	CATEGORIES	TICK	YES (codes)	NO (codes)
**42.**	From fellow workers		1	0
**43.**	From politicians		1	0
**44.**	From Religious leader		1	0
**45.**	On Radio		1	0
**46.**	On Television		1	0
**47.**	From Health Officers		1	0
**48.**	Other (specify		1	0

49. Is there no need for Pap smear screening after receiving HPV vaccination? **(Yes =1, No = 0, I don't know=2)**

- Yes
- No
- I don't know

SECTION 3: Attitude towards the HPV vaccines
<u>Tick the most applicable answer.</u>

	Variable	Yes=1	No=0	I don't know=2

**50.**	Do you believe that your child is at risk of HPV infection?			
**51.**	Do you believe that HPV infection is severe?			
**52.**	Do you believe that the HPV vaccine is effective?			
	Do you want to be educated more on HPV?			

SECTION 4: HPV vaccination willingness (Yes=1, No=0)

53. Would you recommend that your child or a close relative be vaccinated?

- ☐ Yes
- ☐ No
- ☐ I don't know.

54. Would you recommend that young girls (below 10 years) be given the HPV vaccine?

- ☐ Yes
- ☐ No
- ☐ I don't know

SECTION 5: Reasons for vaccine acceptance and hesitancy.
What are the reasons that make you accept the HPV vaccination?

<u>Choose all the responses you deem relevant.</u>

	**Reasons for accepting HPV vaccination**	**Yes=1**	**No= 0**	**I don't know= 3**
**55.**	I know that the vaccine is effective at preventing Cervical cancer			
**56.**	Other parents are getting the vaccine for their daughter and I feel I should do the same for mine			
**57.**	There are verified sources of information declaring the vaccine safe			
**58.**	The doctors recommend the vaccine			
**59.**	The school and government recommend the vaccine			
**60.**	My daughter has been asked to have the vaccine			

What are the reasons why you may be hesitant to have the child vaccinated?

<u>Choose all the responses you deem relevant.</u>

	**Reasons for accepting HPV vaccination**	**Yes=1**	**No= 0**	**I don't know= 3**
**61.**	I am against all Vaccinations			
**62.**	Child refusal			
**63.**	The Vaccine is not safe			
**64.**	Since it's a new vaccine its side effects are not well known			
**65.**	The safety profile of the vaccine is still unknown			
**66.**	There may be lifelong health problems from the vaccine.			
**67.**	The vaccine could result in some short term side effects			
**68.**	The vaccine will result in pain especially when being injected site.			

**69.**	My daughters are not yet of age to receive the vaccine			
**70.**	The vaccine may make the young girls start having sex			
**71.**	I would not want my daughters stigmatized as promiscuous.			
**72.**	My religion does not allow vaccination			
**73.**	The HPV vaccine is not necessary			
**74.**	The HPV vaccine costs. {travel, and others			
**75.**	I have inadequate information about the vaccine to decide.			

For the following section, please indicate whether you agree or disagree with the statement.

		**Agree=1**	**Neutral=2**	**Disagree=0**
**76**	All girls aged 10 years should get the HPV vaccine			
**77**	HPV Vaccine is safe			
**78**	HPV infection is common in Kenya			
**79**	I have enough information about HPV vaccine to guide my daughters			
**80**	I would like to know more about the HPV vaccine.			
**81**	Girls aged 10 years should get education about sex.			

SECTION 6: Decision process.

82. Would you ask your child for permission before you take them for vaccination??**(Yes =1, No = 0, I don’t know=2).**

- Yes
- No
- I don’t know.

END OF QUESTIONNAIRE

Thank you for your time.
